# Quercetin suppresses inflammation by reducing ERK1/2 phosphorylation and NF kappa B activation in Leptin-induced Human Umbilical Vein Endothelial Cells (HUVECs)

**DOI:** 10.1186/1756-0500-6-275

**Published:** 2013-07-16

**Authors:** Mochamad Rasjad Indra, Satuman Karyono, Retty Ratnawati, Safarina G Malik

**Affiliations:** 1 Department of Physiology, Faculty of Medicine, Brawijaya University, Jl. Veteran, Malang 65145, East Java, Indonesia; 2 Eijkman Institute for Molecular Biology, Jl. Diponegoro 69, Jakarta 10430, Indonesia

**Keywords:** Leptin, Quercetin, Ob-Ra, ERK1/2, NFκB, TNFα, HUVECs

## Abstract

**Background:**

High concentrations of plasma leptin and the release of pro-inflammatory cytokines in leptin-resistance in obesity have been reported to trigger endothelial dysfunction. The objective of this study was to elucidate the role of quercetin in modulating leptin-induced inflammation as assessed by the levels of Ob-Ra expression, ERK1/2 phosphorylation, NF-kappa B activation and TNF-alpha secretion in umbilical vein endothelial cells (HUVECs) *in vitro*.

**Findings:**

HUVECs were exposed to either control levels (0 ng/ml) or 500 ng/mL leptin (L) for 48 hours, followed by control or 125 uM quercetin (Q) for another 6 h. The experimental groups were as follows: L0Q0, L0Q125, L500Q0, L500Q125. The presence of the short chain leptin receptor isoform Ob-Ra in HUVECs was determined by Western blot and immunocytochemistry analyses. Ob-Ra expression, ERK1/2 phosphorylation, NF-kappa B activation and TNF-alpha secretion were quantified by ELISA, and NF-kappa B activationby immunofluorescence staining. Our results showed that Ob-Ra expression, ERK1/2 phosphorylation and NF-kappa B activation increased significantly after 500 ng/mL leptin exposure (1.8x, 1.5x, 6.2x for Ob-Ra, ERK1/2 and NF-kappa B, respectively), but were reduced by addition of 125 uM quercetin (0.7x, 0.3x and 0.4x for Ob-Ra, ERK1/2 and NF-kappa B, respectively), and that quercetin could also partially suppress leptin-induced TNF-alpha secretion (3.8x) by 0.8x.

**Conclusion:**

Exposure of HUVECs to leptin up-regulated Ob-Ra expression and elevated ERK1/2 phosphorylation and NFkB activation, and increased TNF-alpha secretion. These effects strongly suppressed by quercetin, with the exception of TNF-alpha which was partially suppressed. The findings might be of clinical significance, as endothelial dysfunction that could lead to cardiovascular disease is preventable, and quercetin is a natural compound found in various plants and fruits.

## Findings

Obesity has become a global health problem, with the prevalence of overweight and obesity reaching critical levels throughout the world, including Indonesia. A national survey in 2007 in 12 Indonesian provinces showed that 18.8% of the population older than 15 years old are obese [1]. Obesity is a major risk factor for cardiovascular disease, hypertension, dyslipidemia and diabetes mellitus, all of which reduce both the quality of life and life expectancy. Obesity is associated with excessive adipose tissue accumulation due to excessive energy intake and insufficient energy expenditure [2], and is characterized by the alteration of leptin levels, a cytokine produced by adipocytes and one of the regulators of energy metabolism. Studies have shown that most obese patients are leptin resistant, and high leptin levels were observed in these individuals [3]. An association between leptin and increased cardiovascular risk has been reported [4], and is associated with increased levels of inflammatory factors exhibiting pro-atherogenic effects [5-7]. Obesity has also been considered as a state of low-grade inflammation [8]; previous research has shown that atherosclerosis is the result of chronic inflammation, and early atherosclerosis formation is induced by pro-inflammatory cytokines and other proteins produced by inflammatory cells [9,10].

In obesity-related high plasma leptin conditions, inflammation occurs when signal transduction pathways are activated, such as activation of NFκβ, by the binding of leptin to its receptor (Ob-R), and subsequent release of the inflammation factors, for instance tumour necrosis factor alpha (TNFα) [11]. Our preliminary results revealed that 500 ng/ml leptin decreases cell proliferation and increases TNFα, monocyte chemoattractant protein-1 (MCP-1), and intracellular Ca^2+^ levels in human umbilical vein endothelial cells (HUVECs) [12].

Quercetin, a flavonoid compound found in plants and fruits, has been reported to have anti-inflammatory effects [13], which are mediated through the inhibition of pro-inflammatory cytokines [14]. The aim of this study was to investigate the effect of quercetin in modulating the expression of Ob-Ra, phosphorylation of ERK1/2, activation of NFκB and secretion of TNFα in leptin-induced human umbilical vein endothelial cells (HUVECs) *in vitro*.

## Methods

### Samples

Human umbilical vein endothelial cells (HUVECs) were obtained from umbilical cords of patients that have undergone cesarean section in Dr. Syaiful Anwar Hospital, Malang, after obtaining informed consent. This research was approved by the institutional research ethical committee from the Faculty of Medicine, Brawijaya University, Malang.

### Cell culture and treatment

HUVECs were isolated and cultured as described previously [15,16]. Briefly, HUVECs were cultured in M-199 medium (Sigma-Aldrich, USA) supplemented with 10% fetal calf serum (Biochrom, Germany), 0.0292 g/mL L-glutamine (Gibco, USA), 50 U/mL penicillin (Gibco, USA), and 50 mg/mL streptomycin (Gibco, USA), at 37°C in a 5% CO2 incubator. Human recombinant leptin (500 ng/mL; Sigma-Aldrich, USA) dissolved in dimethyl sulfoxide/DMSO (MPBio, USA) was added to HUVECs and incubated for 48 hours. Quercetin (125 μM; Sigma-Aldrich, USA) dissolved in methylcellulose (MPBio, USA), was added to leptin-exposed HUVECS for 6 hours. HUVECs that were not exposed to leptin were treated with 0 μM and 125 μM quercetin for 6 hours. Quercetin concentration of 125 μM was reported to have marginal cell toxicity [17].

The experimental groups were: L0Q0 without leptin and quercetin (control, with DMSO and methylcellulose only); L0Q125 without leptin but with 125 μM quercetin; L500Q0 with 500 ng/mL leptin but without quercetin; L500Q125 with 500 ng/mL leptin and 125 μM quercetin. The experiments were repeated 5 times for each group.

### Western blot analysis

Proteins were extracted from HUVECs by using mammalian protein extraction reagent (Pierce, IL). Protein concentrations were determined using Coomassie protein reagent (Bio-Rad, CA). Thirty micrograms of total protein was loaded per lane and separated by 7.5% sodium dodecyl sulfate-Tris-glycine polyacrylamide gel electrophoresis. Proteins were transferred to nitrocellulose membranes and blocked overnight in Tris-buffered saline (TBS) containing 0.1% Tween and 5% nonfat dry milk. Membranes were probed with rabbit polyclonal antibody directed against either human short leptin receptor (ObRa) or human phosphorylated ERK1/2 or human p65 NFκB antibodies (1:1000; Cell Signaling, MA), or mouse antihuman β-actin monoclonal antibody (1:1.000; Santa Cruz Biotech, USA), then were detected using Biotin-conjugated goat anti-rabbit IgG (Santa Cruz Biotech, USA). Visualization was done after incubation with streptavidin-alkaline phosphatase conjugate (Invitrogen, USA), by employing colorimetric detection using nitroblue tetrazolium/5-bromo-4-chloro-3-indolylphosphate reagent (Roche, IN).

### Ob-Ra Immunocytochemistry

HUVECs were fixed with methanol in glass slides, then gently rinsed with phosphate buffer solution (PBS). Normal human serum (1:10 dilution) (MPBio, USA) was applied and incubated for 20–30 minutes at 37°C. Rabbit anti-human Ob-Ra antibody (1:100) (Santa Cruz Biotech, USA) was applied and the specimens were incubated overnight at 4°C. Labelled anti rabbit IgG-SA-HRP (KPL, USA) was applied for 60 minutes, then substrate-chromogen solution 3,3′-diaminobenzidine was added, and the specimens were incubated for 10 minutes. Hematoxylin counterstaining was performed to stain the nucleus. The slides were then covered with cover slips.

### Ezyme linked immunosorbent assay

Cell lysate were obtained from harvested HUVECs, then ELISA was performed to quantify the level of Ob-Ra (BioSource International, USA), total phosphorylated ERK1/2 (Assay Designs, USA), and p50/p65 NFκB (Imgenex, USA).

ELISA for secreted TNFα (Bender MedSystem, Austria) was conducted using the supernatant of HUVECs culture.

### NFκB Immunofluorescence

Sub-confluent HUVECs grown on cover slips were fixed with methanol at room temperature for 10 minutes, labeled with 10 μg/mL antihuman p50/p65 NFκB antibody (Thermo Scientific, USA) for 1 hour, washed 3 times with PBS, then incubated with 8 μg/mL FITC-labeled goat anti-rabbit IgG (Santa Cruz Biotechnology Inc, USA) for 1 hour. Bound antibody was detected using Fluo view 1000 confocal microscope (Olympus, Japan) equipped for epiluminescence.

### Statistical analyses

Results were the average of at least five repeats. Differences between groups were determined by ANOVA, followed by Tukey’s test (SPSS vs. 17). All data were shown as means ± SD, and *p* < 0.05 was considered statistically significant.

## Results

### Ob-Ra was expressed in HUVECs

**Figure 1:**
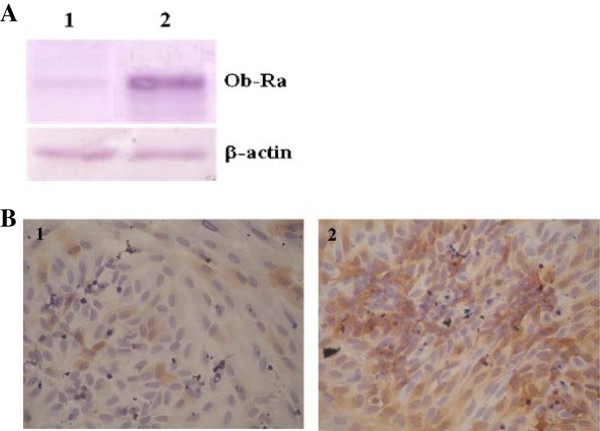
**Ob-Ra was expressed in HUVECs. (A)** Western blot analysis of Ob-Ra in HUVECs showed that exposure of 500 ng/mL leptin for 48 h increased Ob-Ra expression (without (lane 1) and with (lane 2) leptin exposure). β-actin expression in leptin-induced HUVECs was similar with control (without leptin induction). **(B)** Confirmation of increased Ob-Ra expression after leptin exposure by immunocytochemistry using specific antibody against Ob-Ra (without (panel 1) and with (panel 2) leptin exposure).

The short isoform of leptin receptor (Ob-Ra) was expressed in HUVECs, as demonstrated by Western blot analysis shown in Figure 1A. After leptin induction the expression of Ob-Ra was increased almost two folds as compared to control (band intensities are 1488 ± 355.4 vs. 2586 ± 380.8, without and with leptin exposure, *p* = 0.001). Immunocytochemistry staining confirmed the increased expression of Ob-Ra in leptin-induced HUVECs (Figure 1B).

### Quercetin down-regulated leptin receptor expression

**Figure 2:**
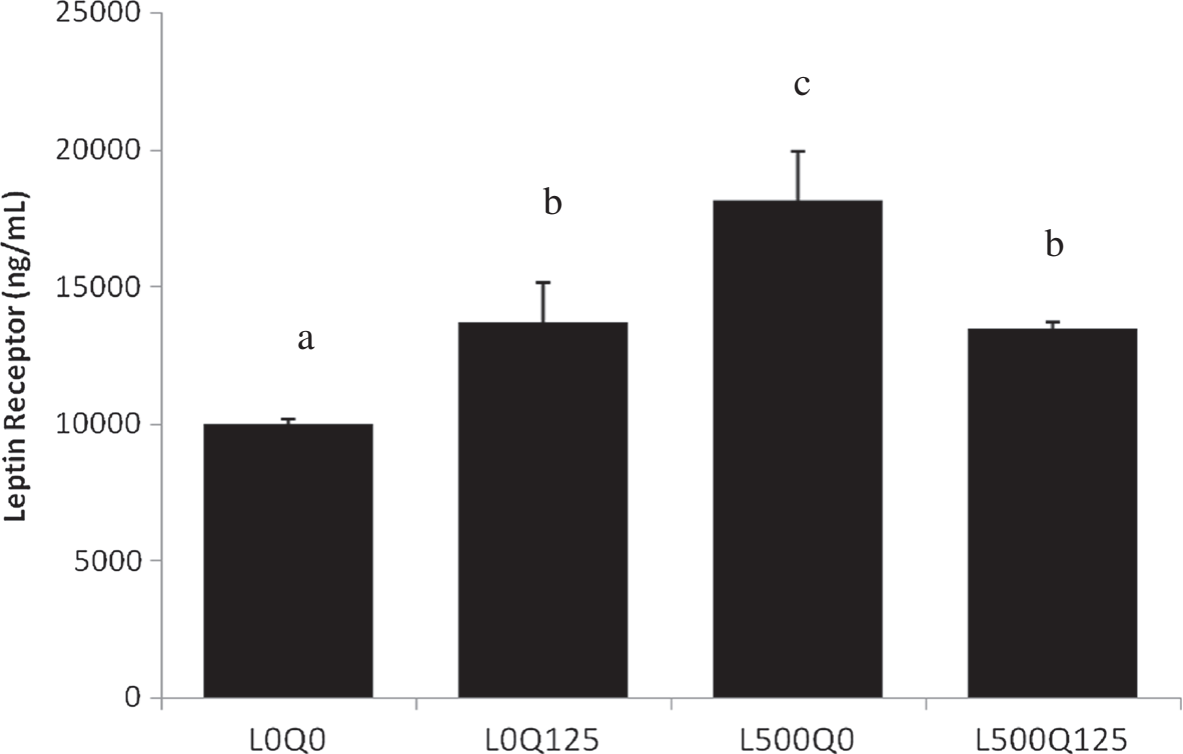
**Quercetin down-regulates leptin receptor expression.** Ob-Ra expression measured by ELISA was increased after 500 ng/mL leptin exposure, and 125 μM quercetin addition was able to decrease the expression of Ob-Ra in leptin-induced HUVECs. L0Q0 control HUVECs without leptin and quercetin; L0Q125 without leptin, but with 125 μM quercetin; L500Q0 with 500 ng/mL leptin, but without quercetin; L500Q125 with 500 ng/mL leptin and 125 μM quercetin. Means without a common letter differ, at least *p* < 0.05.

The short isoform of leptin receptor (Ob-Ra) expression was significantly increased 1.8x after 48 hours leptin exposure (500 ng/ml) (9966 ± 194.0 vs. 18116 ± 1823 ng/mL, L0Q0 vs. L500Q0, *p* = 10^-4^). Quercetin (125 μM) down-regulated the leptin-induced Ob-Ra expression by 0.7x(13449 ± 235.3 vs. 18116 ± 1823 ng/mL, L500Q125 vs. L500Q0, *p* = 0.028). Quercetin alone (L0Q125) increased leptin receptor expression (13660 ± 1452 vs. 9966 ± 194.0 ng/mL, L0Q125 vs. L0Q0, *p* = 0.020) (Figure 2).

### Quercetin reduced leptin-induced ERK1/2 phosphorylation

**Figure 3:**
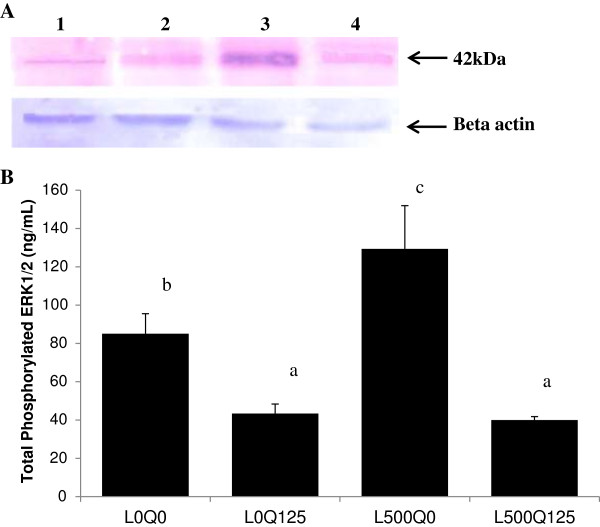
**Quercetin reduced leptin-induced ERK1/2 phosphorylation. (A)** Western blot analysis of phosphorylated ERK1/2 in HUVECs showed that 500 ng/mL leptin exposure for 48 h increased ERK1/2 phosphorylation (without (lane 1) and with (lane 3) leptin exposure). Quercetin alone did not show any effect (lane 2), nevertheless in leptin-induced HUVECs, ERK1/2 phosphorylation was reduced (lane 4). **(B)** Total phosphorylated ERK1/2 level measured by ELISA was reduced after quercetin treatment on leptin-induced HUVECs. L0Q0 control HUVECs without leptin and quercetin; L0Q125 without leptin, but with 125 μM quercetin; L500Q0 with 500 ng/mL leptin, but without quercetin; L500Q125 with 500 ng/mL leptin and 125 μM quercetin. Means without a common letter differ, at least *p* < 0.05.

Quantitative measurement by ELISA revealed a 1.5x higher total phosphorylated ERK1/2 level in leptin-induced HUVECs (85.00 ± 10.44 vs. 129.3 ±22.55 ng/mL, L0Q0 vs. L500Q0, *p* = 0.012). Addition of 125 μM quercetin (L500Q125) reduced ERK1/2 phosphorylation in leptin-induced HUVECs by 0.3x (40.00 ± 1.73 vs. 129.3 ±22.55 ng/mL, L500Q125 vs. L500Q0, *p* = 10^-4^). We observed that quercetin alone also reduced ERK1/2 total phosphorylation (43.33 ± 4.93 vs. 85.00 ± 10.44 ng/mL, L0Q125 vs. L0Q0, *p* = 0.016) (Figure 3).

### Quercetin inhibited leptin-induced NFκB activation

**Figure 4:**
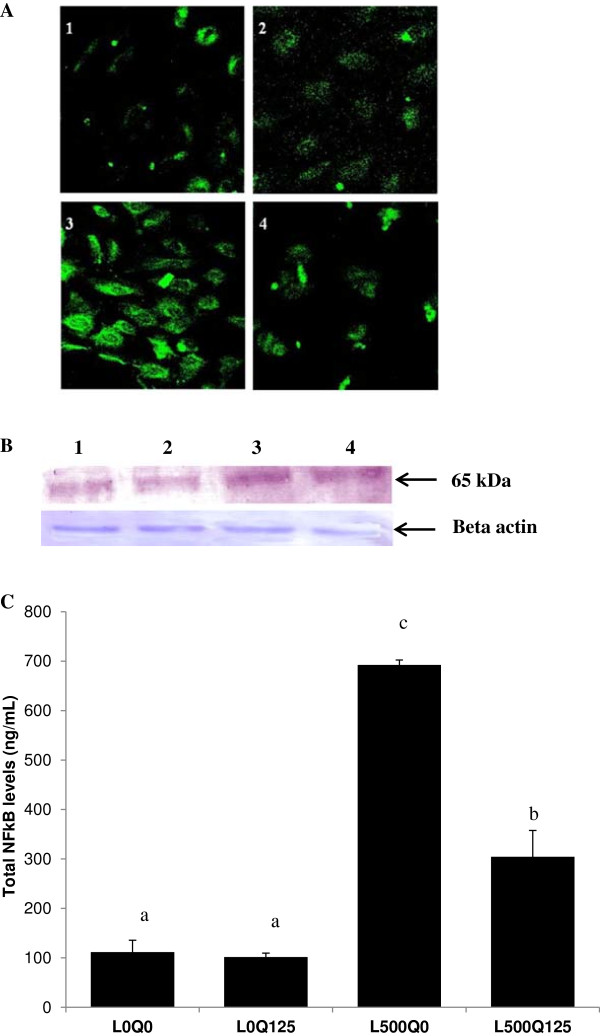
**Quercetin inhibits leptin-induced NFκB activation. (A)** FITC immunofluorescence labeling showed the signal density was increased after 500 ng/mL leptin exposure as compared to control. Quercetin addition in leptin-induced HUVECs reduced the density of NFκβ. Panel 1 control HUVECs without leptin and quercetin; panel 2 without leptin, but with 125 μM quercetin; panel 3 with 500 ng/mL leptin, but without quercetin; panel 4 with 500 ng/mL leptin and 125 μM quercetin. **(B)** Western blot analysis of p65 NFκB in HUVECs confirmed the immunofluorescence labeling. L0Q0 control HUVECs without leptin and quercetin; L0Q125 without leptin, with 125 μM quercetin; L500Q0 with 500 ng/mL leptin, without quercetin; L500Q125 with 500 ng/mL leptin and 125 μM quercetin. **(C)** ELISA measurement showed a significant increased of NFκβ activation in leptin-induced HUVECs when compared to control, and quercetin administration significantly reduced the activated NFκB level. L0Q0 control HUVECs without leptin and quercetin; L0Q125 without leptin, but with 125 μM quercetin; L500Q0 with 500 ng/mL leptin, but without quercetin; L500Q125 with 500 ng/mL leptin and 125 μM quercetin. Means without a common letter differ, *p* < 0.001.

FITC immunolabeling was used to determine the NFκB activation. As shown in Figure 4A signal density was increased after HUVECs was exposed to 500 ng/mL leptin as compared to control. Quercetin alone did not induce NFκB activation. However, quercetin addition in leptin-induced HUVECs reduced the density of NFκβ. This phenomenon was confirmed by Western blot analysis (Figure 4B). A further confirmation by ELISA showed a significant 6.2x increased of NFκβ activation in leptin-induced HUVECs when compared to control (111.6 ± 24.35 vs. 692.4 ± 9.849 ng/mL, L0Q0 vs. L500Q0, *p* = 10^-5^). After quercetin addition, NFκB activation was significantly reduced by 0.4x (304.4 ± 53.33 vs. 692.4 ± 9.849 ng/ml, L500Q125 vs. L500Q0, *p* = 10^-4^). Quercetin alone did not activate NFκB (Figure 4C).

### Quercetin partially suppressed leptin-induced TNFα secretion

**Figure 5:**
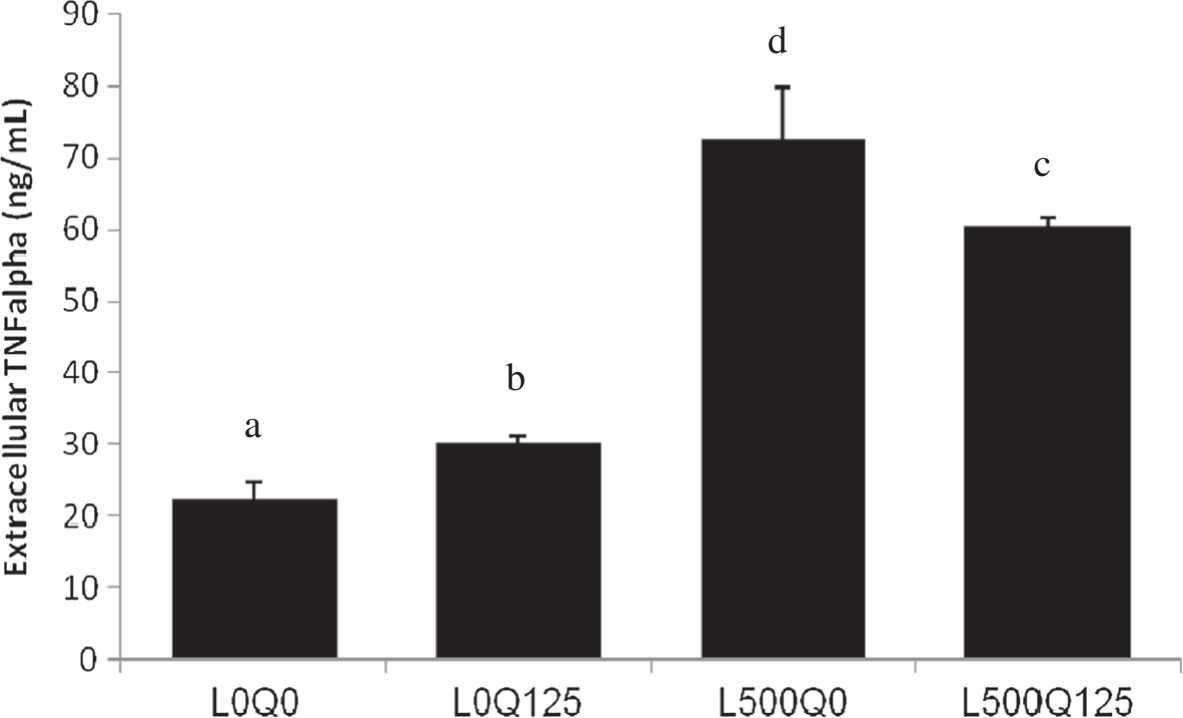
**Quercetin partially suppressed leptin-induced TNFα secretion.** TNFα secretion was markedly increased in leptin-induced HUVECs as compared to control, and was partially decreased after quercetin addition. L0Q0 control HUVECs without leptin and quercetin; L0Q125 without leptin, but with 125 μM quercetin; L500Q0 with 500 ng/mL leptin, but without quercetin; L500Q125 with 500 ng/mL leptin and 125 μM quercetin. Means without a common letter differ, at least *p* < 0.05.

Secreted TNFα level was markedly increased by 3.2x in leptin-induced HUVECs as compared to control (22.39 ± 2.29 vs. 72.50 ± 7.33 ng/mL, L0Q0 vs. L500Q0, *p* = 10^-4^). TNFα secretion was decreased, albeit partially (0.8x), after quercetin addition in leptin-induced HUVECs (L500Q125) when compared to leptin-induced HUVECs without quercetin (L0Q125) (60.38 ± 1.04 vs. 72.50 ± 7.33 ng/mL, L500Q125 vs. L500Q0, *p* = 0.022). TNFα secretation was also increased when HUVECs were exposed to quercetin alone (Figure 5).

## Discussion

We have demonstrated the presence of the short chain leptin receptor isoform Ob-Ra in HUVECs, confirming previous studies [16,17]. We were able to show that Ob-Ra expression was increased after leptin exposure. Leptin has been reported to differentially modulate the expression of its receptors in both dose- and tissue-dependent manner [18]. When treated with high leptin concentration, the level of leptin receptor was increased, reported to be due to changes in receptor number that occur prior to gene expression changes [19], which was expected to happen post translationally [20].

We observed that up-regulated Ob-Ra expression was followed by elevated ERK1/2 phosphorylation, in line with previous reports that demonstrated ERK1 and ERK2 activation by the short leptin receptor isoform [21], and leptin-stimulated pro-inflammatory cytokine release abrogation by ERK 1/2 MAPK inhibitor U0126 [22]. As shown from our result, the elevated ERK1/2 phosphorylation was followed by increased NFκB activation and TNFα secretion, which was in agreement with a previous report that indicated leptin has pro-inflammatory action, involving pro-inflammatory cytokines TNFα through NFκB regulation [22].

Quercetin is one of the most widely distributed flavonoids in fruits, vegetables, tea and wine [23], reported to have anti-inflammatory properties, and might act as health-promoting substances [24,25]. In this study, we showed that 125 μM quercetin was able to down-regulate Ob-Ra expression, reduce ERK1/2 phosphorylation, decrease NFκB activation, and partially suppress TNFα secretion in leptin-induced HUVECs. The anti-inflammatory effect of quercetin has been described in various reports, among others were the ability to suppress ERK phosphorylation in 3 T3-L1 adipocytes [26] and NCI-H292 cells [27], inhibit NFκB activation in murine J774 macrophages [28] and bone marrow-derived macrophages [29], VAT TNFα production in obese Zucker rats [30], reduce the activation of phosphorylated ERK kinase strongly in LPS-induced RAW 264.7 cells and inhibit NFκB activation through both stabilization of NFκB/IκB complex and suppression of proinflammatory cytokines including TNFα [31]. In PBMC, a 50% reduction of TNFα gene expression was observed after 24 h exposure to 50 μM quercetin [14]. A meta-analysis of long-term placebo-controlled human intervention trials reported that TNFα levels were decreased after flavonoid consumption, but only in a fixed model, and a higher dose or a longer duration intervention were not associated with a greater effect size [32]. Although preliminary, our data suggested that suppression of TNFα secretion by quercetin was associated with inhibition of NFκB activation, as reported previously [28]. However, our result showed that quercetin suppression of TNFα secretion appeared to be partial, which we believe might be due to the short duration of quercetin exposure period. Although essential, unfortunately we can not perform further confirmation of TNFα suppression by TNFα mRNA assessment, since our samples were not prepared and stored for RNA analysis. This is the limitation of our study, besides that we only apply one quercetin concentration in our study, which might not be the optimum concentration. Further studies, such as measurements of TNFα mRNA and application of NFkB activation inhibitor, are still needed to clarify this discrepancy.

The suppression of inflammation by quercetin may have clinical significance in preventing cardiovascular disease induced by leptin-resistant in obesity. As reported previously, quercetin administration to obese Zucker rats improved dyslipidemia, hypertension, and hyperinsulinemia, all of which are cardiovascular risk factors [30]. A deeper assessements on the molecular mechanisms of quercetin action in suppressing leptin-induced inflammation are needed as high intake of plant-derived food rich in quercetin or the use of supplements of this flavonoid might be protective and could reduce cardiovascular risks. It might also be a promising area for the development of a flavonoid-based neutrapharmaceutical agents for the treatment of chronic inflammatory disease.

## Conclusion

Leptin exposure of HUVECs resulted in up-regulated Ob-Ra expression and elevated ERK1/2 phosphorylation and NFkB activation, and increased TNF-alpha secretion. Despite strong suppression effect of quercetin on the increased Ob-Ra expression, ERK1/2 phosphorylation and NFkB activation, however, TNFα secretion was only partially suppressed. Therefore, further studies are still needed to elucidate the basis of this exception.

## Abbreviations

HUVECs: Human umbilical vein endothelial cells; TNFα: Tumor necrosis factor alpha; Ob-R: Leptin receptor; MAPK: Mitogen activated protein kinase; ERK1/2: Extracellular regulated kinase 1/2; NFκβ: Nuclear factor kappa b; L: Leptin; Q: Quercetin; ELISA: Enzyme linked immunosorbent assay; FITC: Fluorescence isothiocyanate; ANOVA: Analysis of variance.

## Competing interest

The authors declare that they have no competing interest.

## Authors’ contributions

MRI designed the study, performed data analysis, wrote and revised the final manuscript. SK carried out all assays and performed data analysis. RR designed the study, performed data analysis, wrote and revised the final manuscript. SGM provided direction, performed data analysis and revised the final manuscript. All authors have read and approved the final manuscript.
